# Transcriptome analysis of pod mutant reveals plant hormones are important regulators in controlling pod size in peanut (*Arachis hypogaea* L.)

**DOI:** 10.7717/peerj.12965

**Published:** 2022-02-28

**Authors:** Yaqi Wang, Maoning Zhang, Pei Du, Hua Liu, Zhongxin Zhang, Jing Xu, Li Qin, Bingyan Huang, Zheng Zheng, Wenzhao Dong, Xinyou Zhang, Suoyi Han

**Affiliations:** 1 College of Agronomy, Shenyang Agricultural University, Shenyang, China; 2 Henan Academy of Crop Molecular Breeding, Henan Academy of Agricultural Sciences, Zhengzhou, China

**Keywords:** *Arachis hypogaea* L., Pod size, Plant hormone, Transcriptome analysis, Weighted gene coexpression network analysis

## Abstract

Pod size is an important yield-influencing trait in peanuts. It is affected by plant hormones and identifying the genes related to these hormones may contribute to pod-related trait improvements in peanut breeding programs. However, there is limited information on the molecular mechanisms of plant hormones that regulate pod size in peanuts. We identified a mutant with an extremely small pod (*spm*) from Yuanza 9102 (WT) by ^60^Co γ-radiation mutagenesis. The length and width of the natural mature pod in *spm* were only 71.34% and 73.36% of those in WT, respectively. We performed comparative analyses for morphological characteristics, anatomy, physiology, and global transcriptome between *spm* and WT pods. Samples were collected at 10, 20, and 30 days after peg elongation into the soil, representing stages S1, S2, and S3, respectively. The differences in pod size between WT and *spm* were seen at stage S1 and became even more striking at stages S2 and S3. The cell sizes of the pods were significantly smaller in *spm* than in WT at stages S1, S2, and S3. These results suggested that reduced cell size may be one of the important contributors for the small pod in *spm*. The contents of indole-3-acetic acid (IAA), gibberellin (GA), and brassinosteroid (BR) were also significantly lower in *spm* pods than those in WT pods at all three stages. RNA-Seq analyses showed that 1,373, 8,053, and 3,358 differently expressed genes (DEGs) were identified at stages S1, S2, and S3, respectively. Functional analyses revealed that a set of DEGs was related to plant hormone biosynthesis, plant hormone signal transduction pathway, and cell wall biosynthesis and metabolism. Furthermore, several hub genes associated with plant hormone biosynthesis and signal transduction pathways were identified through weighted gene co-expression network analysis. Our results revealed that IAA, GA, and BR may be important regulators in controlling pod size by regulating cell size in peanuts. This study provides helpful information for the understanding of the complex mechanisms of plant hormones in controlling pod size by regulating the cell size in peanuts and will facilitate the improvement of peanut breeding.

## Introduction

Cultivated peanut (*Arachis hypogaea* L., 2*n* = 4*x* = 40, AABB) is one of the most important oil crops for the human diets worldwide (Zhuang et al., 2019). There has been an increasing demand for oil in recent years, highlighting the need for peanut cultivars with high yields (Wan et al., 2017). The peanut yield is directly proportional to pod weight, seed weight, and the number of pods per plant (Gangurde et al., 2020). Pod size is closely associated with pod weight (Luo et al., 2018) and directly affects peanut yield (Chen et al., 2016). However, specific peanut pod/seed sizes are preferred by different sectors of industry—bigger is not always acceptable. A comprehensive understanding of the molecular mechanisms governing the pod size and mining the key regulatory factors are crucial for improving pod-related traits in peanuts.

Fruit size is one of prominent agronomical traits for crop domestication and improvement (Hussain et al., 2020). Many genes and several pathways/mechanisms involved in fruit size have been identified in several plants, such as *Hyp O-galactosyltransferase* (*HPGT1*) in Arabidopsis (Ogawa-Ohnishi & Matsubayashi, 2015), *BnDA1* in rapeseed (Wang et al., 2017), *Grain width 2* (*GW2*) in rice (Yamaguchi et al., 2020), *Salt and drought-induced RING finger1* (*TaSDIR1-4A*) in wheat (Wang et al., 2020), and *KERNEL NUMBER PER ROW 6* (*KNR6*) in maize (Jia et al., 2020). Several genes related to plant hormones have been reported to regulate organ size in different plants. Hussain et al. (2020) summarized the hormone-related genes involved in regulating fruit size and discussed the pathways/mechanisms. In Arabidopsis, the overexpression of the *AUXIN-REGULATED GENE INVOLVEDIN ORGAN SIZE* (*ARGOS*) gene increased cell size and cell number, resulting in enlarged leaves and siliques (Wang et al., 2009). *AUXIN RESPONSE FACTOR 18* (*ARF18*) encodes an auxin-response factor, and disfunction of the *ARF18* gene can induce longer siliques by accelerating cell expansion in the silique wall of *Brassica napus L*. (Liu et al., 2015). *GA 20-oxidases* (*GA20ox*) genes, *GA20ox1* and *GA20ox2*, involved in gibberellin (GA) biosynthesis, regulate hypocotyl, internode, and siliques elongation in Arabidopsis (Rieu et al., 2008). *TaCYP78A3* encodes a cytochrome P450 CYP78A3, and disfunction of the *TaCYP78A3* can cause a decrease in wheat seed size (Ma et al., 2015). The *GRAIN LENGTH2* (*GL2*) is allelic to *GROWTH REGULATION FACTOR4* (*OsGRF4*) and positively regulates brassinosteroid (BR) responses to promote rice grain development (Che et al., 2015).

Several major quantitative trait loci (QTLs) related to peanut pod size have been reported in recent years (Huang et al., 2015; Chen et al., 2016; Luo et al., 2017; Luo et al., 2018; Alyr et al., 2020; Chu et al., 2020; Gangurde et al., 2020). Luo et al. (2017) identified three major QTLs for hundred-pod weight (HPW), pod length and width (PL and PW), which were co-localized in the same region on chromosome A05. Furthermore, Luo et al. (2018) co-localized two major QTLs controlling PL and HPW in the same interval on chromosome A05, and three major QTLs for PL, PW, and HPW in the same interval on chromosome A07. Chu et al. (2020) identified one major QTL for pod-related traits in a 9 Mbp interval on linkage group A05. Alyr et al. (2020) identified one QTL associated with pod and seed size in a 168.37 kb interval on chromosome A07. Gangurde et al. (2020) identified 44 major effect QTLs related to PW and SW in two nested-association mapping populations, and found that most of the QTLs distributed on chromosomes A05, A06, B05, and B06.

Peanut has an unique characteristic of fruit production. It accomplishes flowering and fertilization aerially, but forms the fruits underground (Smith, 1950). After fertilization, the peanut gynophore harboring embryo elongates, and forms a special peg-like structure that eventually pushes the embryo into the soil for underground pod development (Xia et al., 2013). The pre-embryo develops into a globular embryo after approximately 9 days after peg elongation into the soil (DAP) and the pod becomes enlarged (Zhang et al., 2016). Pod development then undergoes rapid growth and expansion during 10–25 DAP. The pod reaches its final size during 25–30 DAP. Previous studies have shown that plant hormones such as auxin (Moctezuma & Feldman, 1998; Moctezuma & Feldman, 1999; Moctezuma, 1999), gibberellin (Lee, Ketring & Powell, 1972; Shushu & Cutter, 1990), cytokinin (Shushu & Cutter, 1990), and ethylene (Shlamovitz, Ziv & Zamski, 1995) play important roles in gynophore elongation and pod development in peanut (Moctezuma, 2003). There has been increased interest in transcriptome research related to pod development in peanut recently (Clevenger et al., 2016; Li et al., 2017; Wan et al., 2017; Sinha et al., 2020; Yang et al., 2020). Clevenger et al. (2016) constructed a developmental transcriptome map using 22 different tissue types and ontogenies, including a complete reproductive series from flower to fully mature seed, and annotated 21 developmental co-expression networks. Wan et al. (2017) conducted transcriptome analyses at three pod developmental stages in peanut and identified two candidate genes related to lignin responsible for pod width. Sinha et al. (2020) provided a comprehensive transcriptome map using 20 diverse tissues, and provided insights on gene expression patterns of seed development, photomorphogenesis, allergens, gravitropism, and oil biosynthesis. Yang et al. (2020) revealed the molecular mechanism of calcium on peanut pod development by using transcriptome analysis. However, the molecular mechanisms of plant hormones regulation pod size in peanut are still not well understood.

In the present study, we identified a mutant with an extremely small pod (*spm*) from Yuanza 9102 (WT) using ^60^Co γ-radiation mutagenesis, and performed morphological, anatomical, physiological, and transcriptome analyses for pods in WT and *spm* at three key pod developmental stages. The comparative analyses revealed significant differences in the pod size, cell size, contents of plant hormones, and dynamic gene expression between WT and *spm*. Pairwise comparisons and WGCNA analyses identified several plant hormone-related genes, which were involved in determining peanut pod sizes. Our study contributes to the understanding of the transcriptome dynamics of peanut pod development and are helpful for elucidating the complex regulatory mechanism of pod size in peanuts.

## Materials & Methods

### Plant materials and RNA sample collection

The peanut *spm* mutant was selected from an ^60^Co γ-radiation-induced population originating from Yuanza 9102 (WT). The *spm* mutant was self-pollinated for 10 generations to ensure a stabilized line for this study. Both WT and *spm* mutant were grown in 2019 in the Yuanyang experiment station of the Henan Academy of Agricultural Sciences. Days to first flower, we marked the flowers and tied the elongating aerial pegs with colored tags (Yin et al., 2013). We then artificially covered the tagged pegs with soil to ensure that all pegs elongation into the soil on the same day. Pods were harvested at 10, 20, and 30 days after peg elongation into the soil, which were designated as stages S1, S2, and S3. Three pods from one plant were pooled as one biological replicate, and three different plants represented three biological replicates. All pods were immediately frozen in liquid nitrogen and then stored at −80 °C for RNA isolation.

### Trait measurements

The pod and seed traits, including length, width, and weight, were measured as described in Jiang, Duan & Ren (2006). A total of 15 naturally matured dry pods and seeds from five individual plants were harvested. A total of 30 pods were sampled from the tagged pegs of ten individual plants from stages S1, S2, and S3, respectively. These pods were then used to measure pod and seed traits.

### Microscopic analyses

Peanut pericarps were collected at stages S1, S2, and S3, and immediately fixed at 4 °C in 2.5% glutaraldehyde in 0.2 M phosphate buffer (pH = 7.3–7.4) for 24 h. Pericarps were dehydrated in an ethanol series (75% for 4 h, 85% for 2 h, 90% for 2 h, 95% for 1 h, and 100% for 1 h). Pericarps were then processed in xylene for 10 min and embedded in paraffin (Chen et al., 2013). The pericarps were cut with a rotary microtome (RM2016, Leica) to a 4 µm thickness. The samples were stained with safranin O/fast green and scanned using NIKON ECLIPSE E100 microscope.

### Plant hormone quantifications

All the pods of *spm* and WT used for indole-3-acetic acid (IAA), BR, and GA quantification were sampled from the same plants used for RNA-seq at stages S1, S2, and S3. A detailed method for pod collection and store was described in the RNA sample collection. The method for extraction, purification, and quantification of the IAA, BR, and GA by enzyme-linked immunosorbent assay kits was modified following the protocol described in Wang et al. (2012). Differences in plant hormones concentrations between *spm* and WT pods were tested using ANOVA.

### RNA-seq and differential genes expression analyses

Total RNA from the marked pod harvested at stages S1, S2, and S3 was extracted using the TRIzol reagent kit (Invitrogen, CA, USA). RNA integrity and quality were tested by using an Agilent 2100 bioanalyzer (Agilent Technologies, CA, USA). Subsequently, a total of 18 libraries were constructed, and the transcriptome sequencing was performed on Illumina HiSeq2500 platform by the Genedenovo Biotechnology Company (Guangzhou, China). The RNA-seq sequencing data in this study have been uploaded to China National Center for Bioinformation under BioProjects, PRJCA006624 (GSA: CRA005103, https://www.cncb.ac.cn/gsa). Low-quality bases and adaptor sequences were eliminated through fastp (v0.18.0; Chen et al., 2018). The remaining sequences were mapped to the ribosome RNA (rRNA) database using Bowtie 2 (v2.2.8; Langmead & Salzberg, 2012), and the rRNA mapped sequences were filtered out. The high-quality sequences were aligned to the tetraploid peanut reference genome Tifrunner.gnm1.KYV3 (Bertioli et al., 2019; https://peanutbase.org) using HISAT (v2.0.0; Kim, Langmead & Salzberg, 2015). Transcripts were assembled and the fragments per kilobase per million reads (FPKM) values were calculated using StringTie (v1.3.1; Pertea et al., 2015). Principal component analysis (PCA) was conducted using ‘prcomp’ utilities in R package. The differential expression between two genotypes at each developmental stage was performed using DESeq2 (Love, Huber & Anders, 2014). The differentially expressed genes (DEGs) with the false discovery rate (FDR) < 0.05 and the absolute fold change (|FC|) ≥ 2 were identified. Gene Ontology (GO), the Kyoto Encyclopedia of Genes and Genomes (KEGG), and MapMan analyses were performed for functional analysis of DEGs as previously described in Dun et al. (2016). The terms/pathways in GO and KEGG analyses with a *q*-value (FDR) < 0.05 were considered to be significantly enriched. The MapMan categories with significance value < 0.05 were used for performing pathway enrichment analysis of DEGs.

### Weighted gene co-expression network analysis (WGCNA)

WGCNA was performed for analyzing gene correlation patterns among different samples using the WGCNA software package as described in Langfelder & Horvath (2008). The highly co-expressed gene modules were inferred with a power β of 8, a minimal module size of 50, and a merge-CutHeight of 0.9 after removing the low-quality genes with unstable effects on the results were removed. Association of modules and the particular samples was evaluated using the module eigengene value. The interaction network of genes in modules was visualized using Cytoscape (v3.7.1; Shannon et al., 2003).

### Quantitative real-time PCR (qRT-PCR)

qRT-PCR analysis was performed using the same pod samples as those used for RNA-seq. The gene-specific primers for qRT-PCR were listed in Table S1. qRT-PCR was conducted using an ABI QS5 (ABI, USA) with SYBR Green detection (TIANGEN, Beijing, China). The actin gene in peanut was used as internal reference for normalizing expression levels of nine genes through the 2^−ΔΔCt^ method (Livak & Schmittgen, 2001).

**Figure 1:**
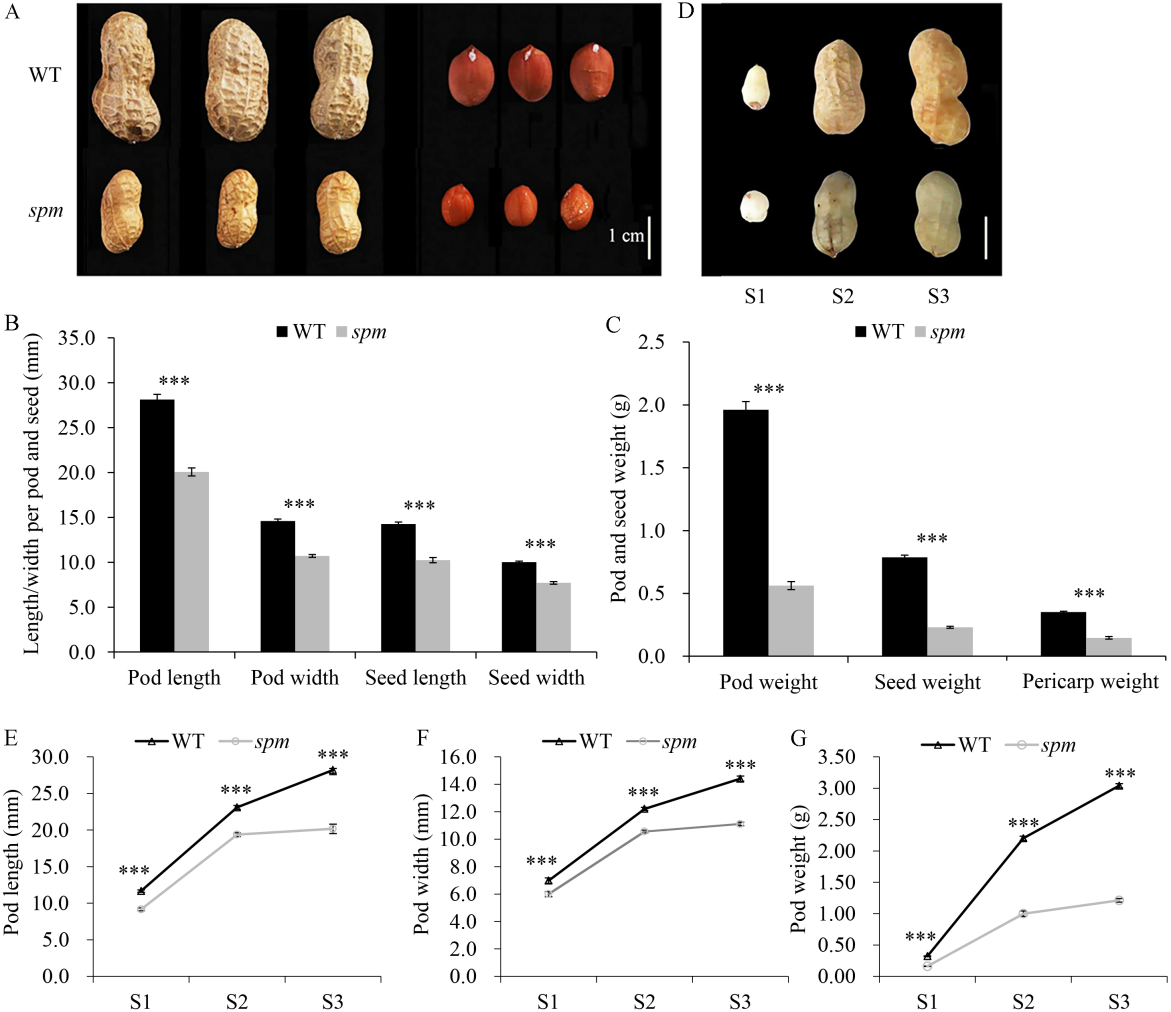
Pod phenotypes at different developmental stages in WT and *spm*. (A) Phenotypes of mature dry peanut pod and seed in WT and *spm*. (B) The mature dry pod and seed length/width in WT and *spm*. (C) The mature dry pod and seed weight in WT and *spm*. (D) Phenotypic characterizations of WT and *spm* pods at three different developmental stages. The samples were collected at 10, 20, and 30 days after peg elongation into the soil (DAP) in WT and *spm*, and the three different developmental stages were designated as stages S1–S3. (E–G) Physical measurements in pod length (E), pod width (F), and pod weight (G) of WT and *spm* at three different developmental stages. The trait values were presented as mean ± SE. The asterisk showed the significant difference between WT and *spm* (*t*-test; *, *P* < 0.05; **, *P* < 0.01; ***, *P* < 0.001).

## Results

### Morphological, anatomical, and physiological analyses on pods at three developmental stages of WT and *spm*

Yuanza 9102 (WT) and its small pod mutant (*spm*), which differ significantly in pod size/weight, were selected to investigate the pod differences (Fig. 1A). The mature dry pod and seed sizes of WT, including length and width, were significantly greater (*P* < 0.001) than those of *spm* (Fig. 1B). The length and width of the pod decreased from 28.12 and 14.60 mm in WT to 20.06 and 10.71 mm in *spm*, respectively. Meanwhile, the length and width of the seed decreased from 14.26 and 10.02 mm in WT to 10.24 and 7.71 mm in *spm*, respectively. The dry weights of the pod, seed, and hull in WT were approximately 3.50-, 3.43-, and 2.50-times as much as those in *spm*, respectively (Fig. 1C). The differences in pod size and weight between WT and *spm* at 10, 20, and 30 DAP were also analyzed. The three stages of pod development were designated as stages S1–S3 (Fig. 1D). Both WT and *spm* pods grew rapidly from stages S1 to S2, but slowly from stages S2 to S3 (Figs. 1E–1G). The rate of increase for pod weight in *spm* was always slower than that in WT from stages S1 to S3 (Fig. 1G). Finally, the pod weight increased 6.56 times in *spm*, but increased 10.69 times in WT from stages S1 to S3. The growth rate for pod size in *spm* was similar to that in WT from stages S1 to S2, but was slower in *spm* than in WT from stages S2 to S3 (Figs. 1E and 1F). The pod size increased 1.20 times and 0.85 times in length and width, respectively, in *spm*, and 1.42 times and 1.06 times in length and width respectively, in WT, from stages S1 to S3. The differences in pod size and weight between WT and *spm* were seen at stage S1 and were even more pronounced at stages S2 and S3 (Figs. 1E–1G). At stage S1, the pod length, width, and weight of WT were 27.65%, 16.53%, and 62.50% more than those of *spm*, respectively. At stage S2, WT showed 19.26%, 15.63%, and 120% increases in pod length, width, and weight compared with *spm*, respectively. At stage S3, the pod length, width, and weight of WT were 39.93%, 29.70%, and 151.24% more than those of *spm*, respectively.

**Figure 2:**
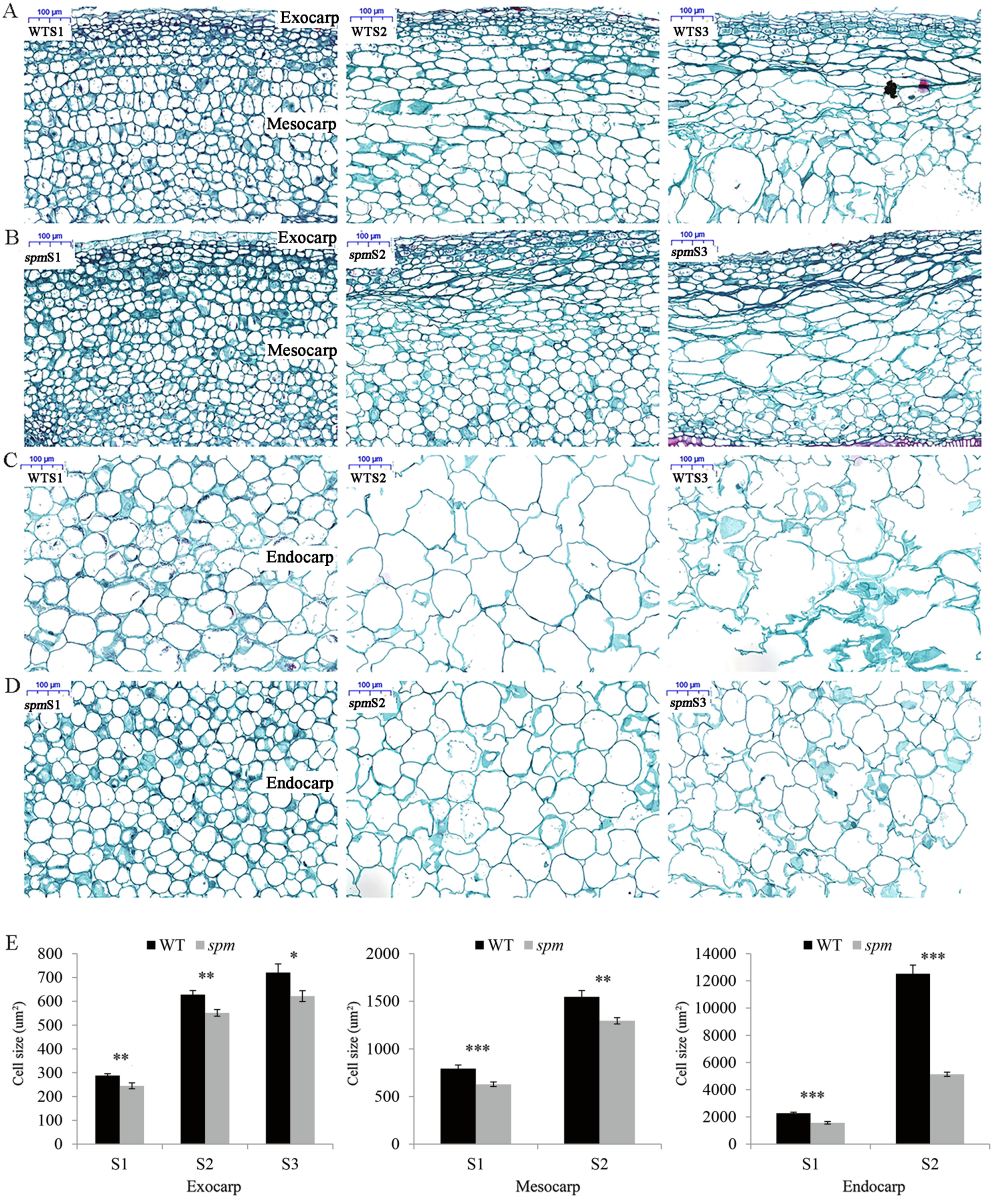
Transverse sections of pods in WT and *spm*. (A–D) The transverse sections of the exocarp, mesocarp, and endocarp in WT and *spm* pods from stages S1–S3. WTS1, WTS2, and WTS3 indicated stages S1, S2, and S3 of WT pods, respectively. Similarly, *spm*S1, *spm*S2, and *spm*S3 indicated stages S1, S2, and S3 of *spm* pods, respectively. (E) The average areas of pod cells in unit view. Three unit views were selected to measure cell areas and values were presented as mean ± SE. The asterisk showed a significant difference between WT and *spm* (*t*-test; *, *P* < 0.05; **, *P* < 0.01; ***, *P* < 0.001).

The anatomical structures of the pod cell, including the exocarp, mesocarp, and endocarp, were analyzed to investigate the cellular differences between WT and *spm*. The results showed that the cell areas increased from stages S1 to S2 in both samples (Figs. 2A–2E). The cell areas were difficult to calculate due to the cell disruption of the mesocarp and endocarp at stage S3. The exocarp cell areas increased approximately 1.50 times in *spm* and WT from stages S1 to S3, the mesocarp cell areas increased approximately 1.00 times in *spm* and WT from stages S1 to S2, and the endocarp cell areas increased approximately 2.28 times and 4.51 times in *spm* and WT from stages S1 to S2, respectively. The exocarp cell areas in *spm* were significantly smaller than those in WT at stages S1 (*P* = 0.007) and S2 (*P* = 0.002). Similarly, the mesocarp cell areas in *spm* were significantly smaller than those in WT at stages S1 (*P* < 0.001) and S2 (*P* = 0.002). The cell areas of *spm* in the endocarp were always significantly smaller (*P* < 0.001) than those of WT from stages S1 to S2 (Fig. 2E). These results suggested that a reduction in cell size may result in decreased pod size in *spm*.

**Figure 3:**
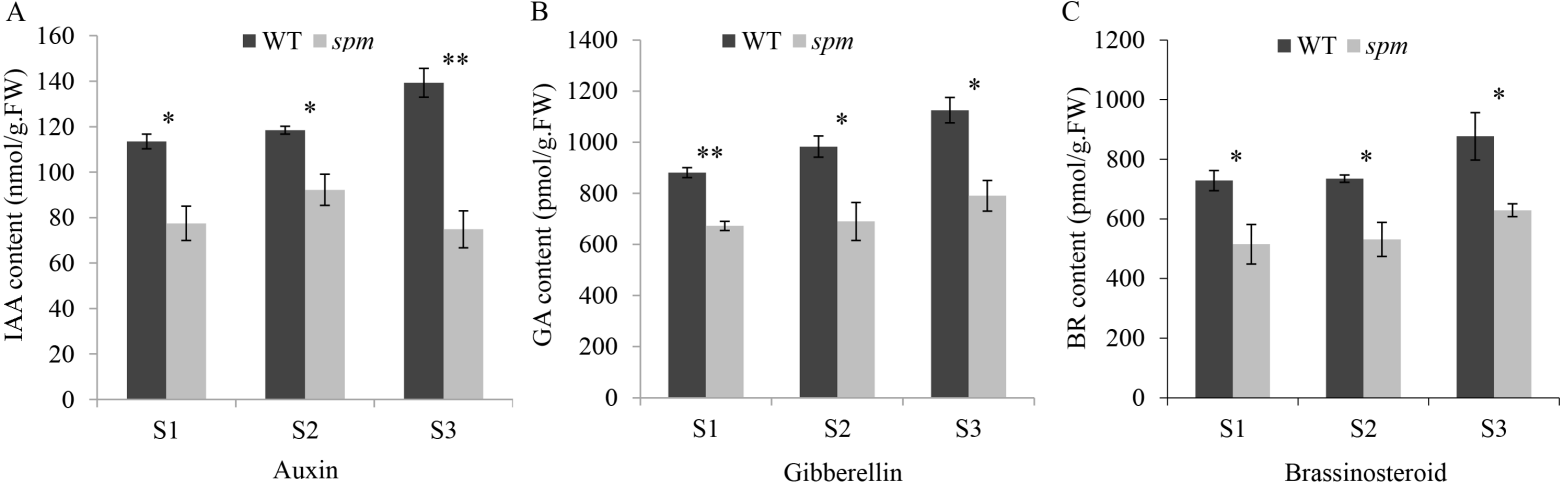
The contents of plant hormones in the *spm* and WT pods from stages S1–S3. (A) The IAA contents in the *spm* pods were significantly lower than in WT pods from stages S1–S3 (S1: *P* = 0.012, S2: *P* = 0.021, S3: *P* = 0.003). (B) The GA contents were significantly lower in the *spm* pods than in WT pods from stages S1–S3 (S1: *P* = 0.001, S2: *P* = 0.026, S3: *P* = 0.013). (C) The BR contents were significantly lower in the *spm* pods than in WT pods from stages S1–S3 (S1: *P* = 0.045, S2: *P* = 0.025, S3: *P* = 0.040). Statistically significant differences were analyzed according to three biological replicates (*t*-test; *P < 0.05; **P < 0.01; ***P < 0.001).

In order to determine the influence of plant hormones on pod size, we measured the contents of several important growth hormones (IAA, GA, and BR) in WT and *spm* pods at three stages. The results showed that the *spm* pods had strikingly lower contents of IAA, GA, and BR than the WT pods at all three stages (Fig. 3), indicating that IAA, GA, and BR may be important for controlling pod size.

### RNA-Seq analyses on pods at three developmental stages of WT and *spm*

**Figure 4:**
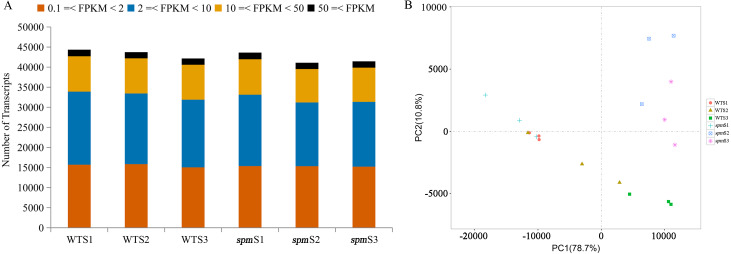
Gene expression profilings of WT and *spm* pods at three developmental stages. (A) Numbers of detected transcripts in each pod sample at different developmental stages in WT and *spm*. (B) Principal component analysis (PCA) plots for pod development at different stages in WT and *spm*.

RNA-Seq analyses were performed at stages S1, S2, and S3 with three independent biological replicates to understand the genetic mechanism of the morphological differences in the pods of WT and *spm*. A total of 18 samples were sequenced, and 35.80–67.65 million high-quality reads were obtained for each sample (Table 1). Approximately 67.83%–97.83% of the high-quality reads were mapped to the peanut tetraploid genome Tifrunner.gnm1.KYV3, and 71,433 genes, including 67,124 known and 4,309 novel genes, were obtained. Genes with FPKM < 0.1 in all samples were removed, and 44,346, 43,737, and 42,146 genes were detected at stages S1, S2, and S3 in WT, respectively. Similarly, 43,606, 41,084, and 41,435 genes were identified at stages S1, S2, and S3 in *spm*, respectively. The number of genes with very high expression levels (FPKM ≥ 50) accounted for approximately 4% in different samples. Approximately 20%, 40%, and 36% of genes with high (10 ≤ FPKM < 50), moderate (2 ≤ FPKM < 10), and low (0.1 ≤ FPKM < 2) expression levels, respectively, were identified in different samples (Fig. 4A).

**Table 1:** Summary of RNA-seq reads for WT and *spm*.

Sample	Stage	Raw data	High-quality reads	Uniquely mapped reads	Total mapped reads
WT-1	S1	50093412	48918128	40473799	47855226
WT-2	S1	69607752	67654356	55076743	65389434
WT-3	S1	44199200	43364394	35244184	41419026
WT-1	S2	42237044	41326932	33873522	39953048
WT-2	S2	47354224	46117086	37815080	44967835
WT-3	S2	44691048	43672758	35843063	42344975
WT-1	S3	43019374	42141746	28862870	34269296
WT-2	S3	41185472	39844756	29440277	35222963
WT-3	S3	45976150	44514214	35417643	42529572
*spm*-1	S1	39160378	37980574	30608769	36814261
*spm*-2	S1	40327090	38061154	30448990	37151385
*spm*-3	S1	42090034	41132622	33867279	40049165
*spm*-1	S2	38999180	37628332	30678611	36728419
*spm*-2	S2	44578766	43264764	29754842	35576995
*spm*-3	S2	45530262	44221988	30884178	36892623
*spm*-1	S3	44145446	43302766	24637836	29372461
*spm*-2	S3	37115942	35796026	28857209	34561356
*spm*-3	S3	43005748	41511116	31935728	38276792

PCA was performed on 18 samples to better understand the transcriptome dynamics of pod development in WT and *spm* (Fig. 4B). The samples from two genotypes at stage S1 were closely grouped but showed somewhat distinct transcriptional activity. Striking differences were observed in WT and *spm* at stage S2. The WT pod transcriptome at stage S2 was closely clustered to the *spm* pod transcriptome at stage S1, whereas stage S2 of the *spm* pod transcriptome tended toward stage S3 of the *spm* pod transcriptome. This indicated that the temporal development progressed faster in *spm* pods than in WT pods. The pods of both genotypes at stage S3 showed striking differences, indicating that substantial differences still existed in their transcriptional programs.

**Figure 5:**
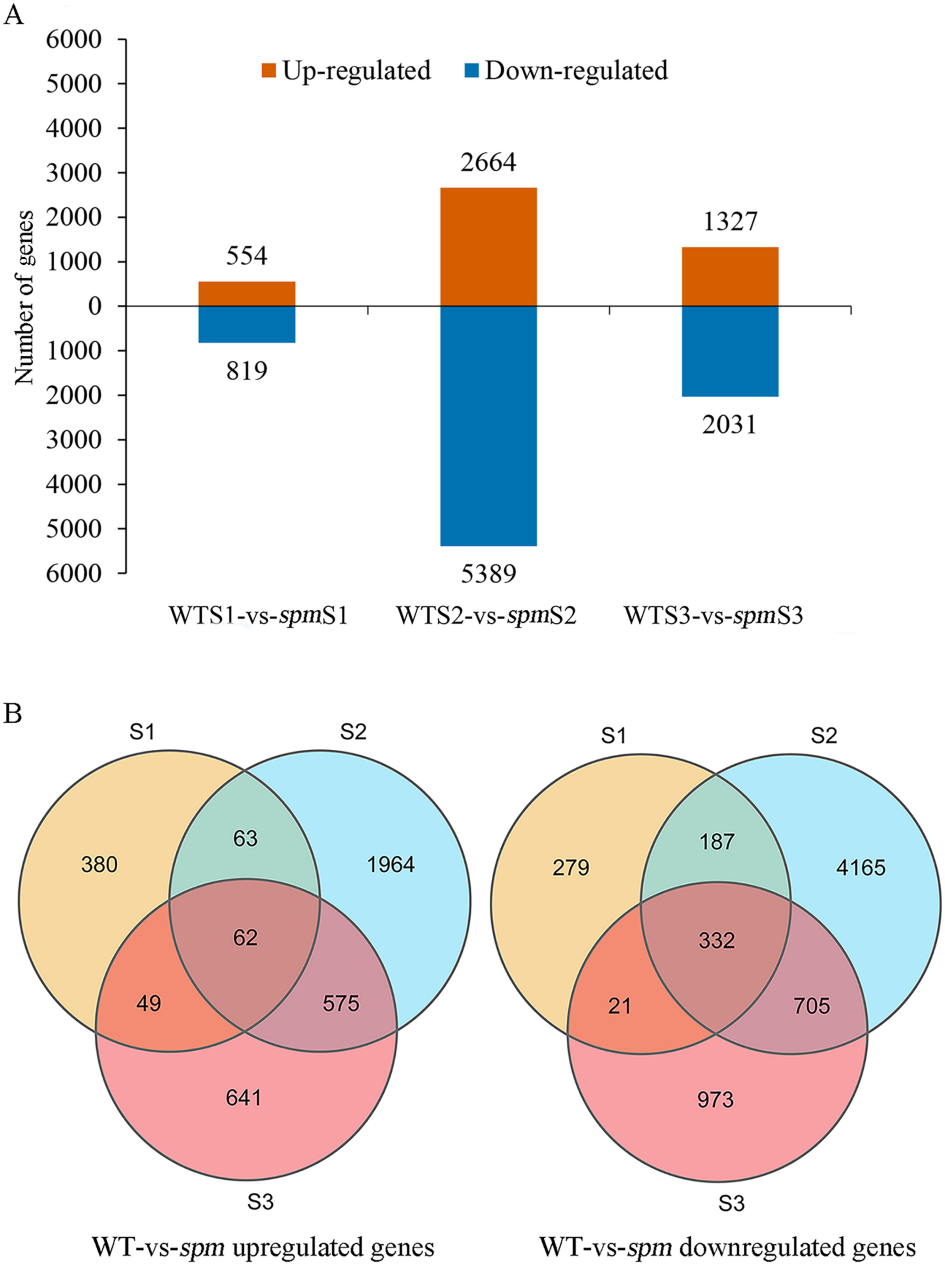
The number of up-regulated and down-regulated genes at each stage of pod development in *spm* as compared with WT. (A) The number of differentially expressed genes from three different stages in WT and *spm*. (B) The overlapping differentially expressed genes between WT and *spm* at three stages.

Pairwise comparison at each developmental stage was performed to investigate the transcriptional differences that characterize different pod size in WT and *spm*. A total of 10,396 DEGs (|FC|≥ 2 and FDR < 0.05) were detected in *spm* pods compared with WT pods (Fig. 5A). Among these DEGs, 3,734 genes were more highly expressed, and 6,662 genes had lower expression levels in *spm* than in WT. At stage S1, 554 genes were up-regulated and 819 genes were down-regulated in *spm* relative to WT. At stage S2, the number of DEGs peaked, and 8,053 DEGs were detected between *spm* and WT, with 2,664 genes up- and 5,389 genes down-regulated in *spm* relative to WT. At S3 stage, 1,327 genes were up-regulated and 2,031 genes were down-regulated in *spm* compared with WT. Only 62 up-regulated genes overlapped among all three developmental stages in *spm* compared with WT; Stage S1 shared 125 and 111 up-regulated genes with stages S2 and S3, respectively; 637 up-regulated genes overlapped between stages S2 and S3 (Fig. 5B). However, 332 down-regulated genes overlapped at all developmental stages in *spm* compared with WT; 519 down-regulated genes were found between stage S1 and S2, and 353 down-regulated genes were identified between stage S1 and S3; 1,037 down-regulated genes overlapped between stages S2 and S3 (Fig. 5B).

**Figure 6:**
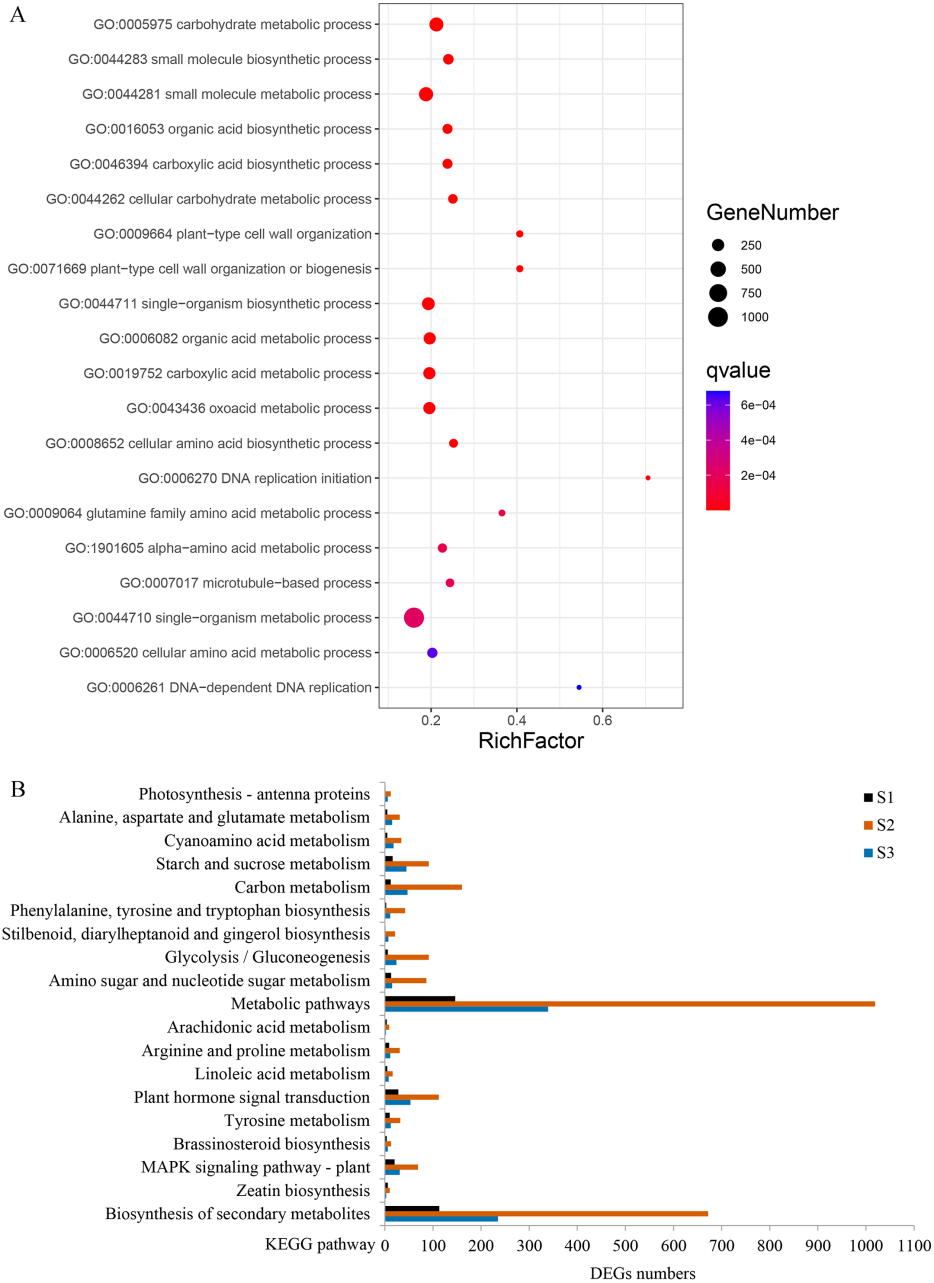
Gene ontology (GO) and Kyoto Encyclopedia of Genes and Genomes (KEGG) enrichment analyses of DEGs between WT and *spm*. (A) The top 20 enriched biological processes for DEGs in the GO enrichment analysis. (B) KEGG pathway enrichment analysis for DEGs at three stages.

GO enrichment analysis revealed that 80 terms were significantly enriched in biological processes. The term ‘carbohydrate metabolic process’ was the most enriched, followed by ‘small molecule metabolic process’, ‘organic acid biosynthetic process’, ‘plant-type cell wall organization’, and ‘single-organism biosynthetic process’ (Fig. 6A, Table S2). Several other biological processes involved in cell wall metabolism were significantly enriched, including, ‘DNA-dependent DNA replication’, ‘microtubule-based process’, ‘cellular glucan metabolic process’, and ‘response to biotic stimulus’ were also significantly enriched (Table S2). KEGG enrichment analysis was performed, and a total of 103, 131, and 124 pathways were enriched at stages S1, S2, and S3, respectively. Many DEGs at stages S2 and S3 were enriched in ‘metabolic pathways’, ‘plant hormone signal transduction’ and ‘starch and sucrose metabolism’; however, the numbers of DEGs in these three pathways were remarkably reduced at stage S1 (Fig. 6B). In the ‘plant hormone signal transduction’ pathway, 112 DEGs were enriched, and most of them were up-regulated in WT compared with *spm* at stage S2. These results indicated that genes involved in ‘plant hormone signal transduction’ have striking differences between WT and *spm* pods at stage S2.

**Figure 7:**
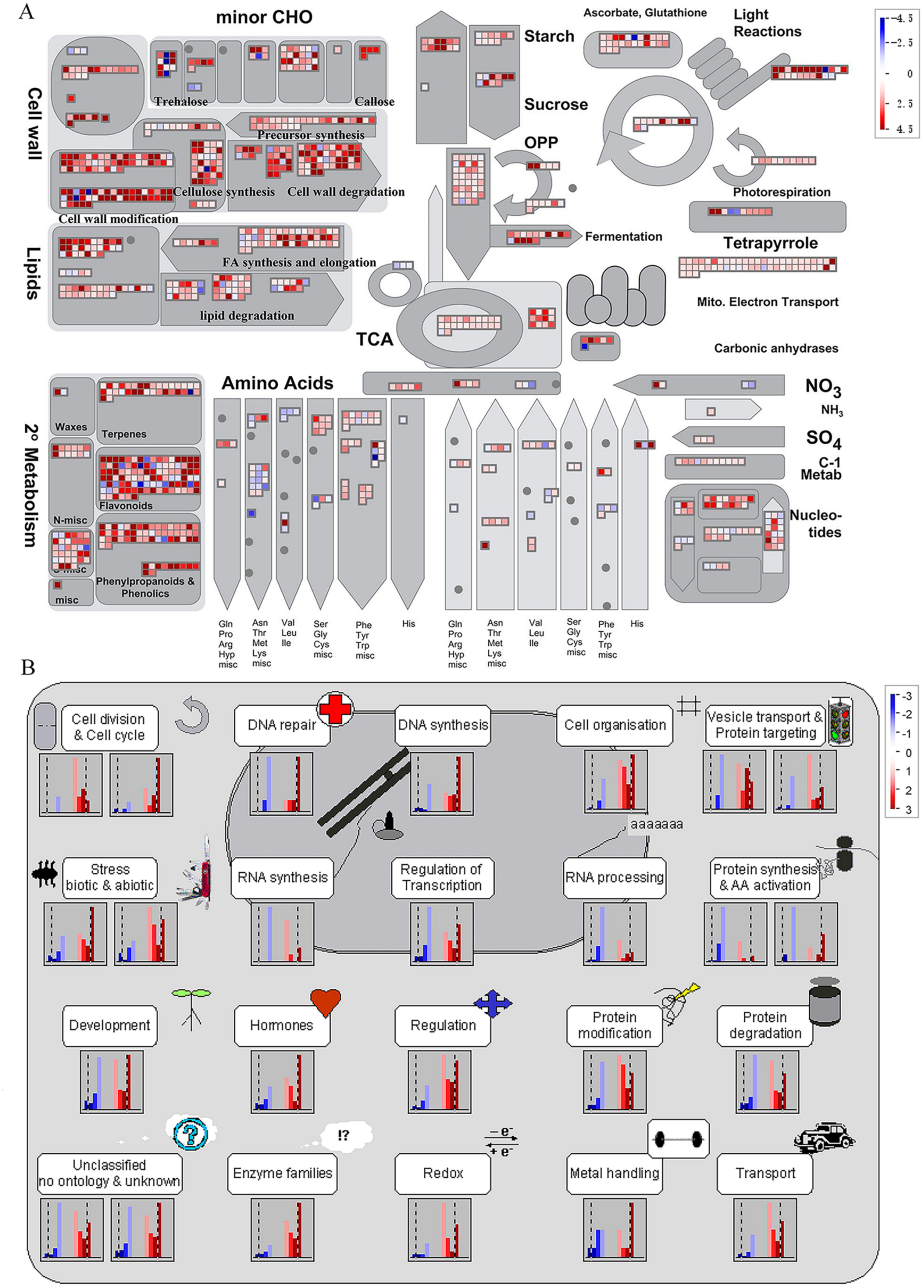
Differential genes expression in *spm* pod as compared with WT pod at stage S2. (A) Metabolic pathways with differential expression profile in *spm* pod as compared with WT pod at S2 stage. (B) Cellular response pathways showing differential expression between *spm* pod and WT pod at stages S2. Differentially expressed genes between *spm* and WT at stage S2 were loaded into MapMan to generate the overview. On the log_2_ scale, dark blue and dark red colors represent higher and lower expression, respectively, in *spm* as compared with WT.

It is noteworthy that a majority of DEGs were identified at stage S2 between WT and *spm*. Furthermore, the numbers of DEGs in the KEGG pathways associated with C and N metabolism peaked at stages S2, indicating that stage S2 may be an important period in pod development. To investigate the metabolic pathways affecting the differences of pod size between WT and *spm* at stage S2, we overlaid the DEGs onto the metabolic pathways using the MapMan. The results showed that the genes in certain metabolic pathways exhibited striking differential expression levels between two genotypes at stage S2 (Fig. 7). For example, the genes involved in starch and sucrose metabolism, cellulose synthesis, cell wall modification, cell division and cell cycle showed higher expression levels in WT (Figs. 7A and 7B), indicating that actively cell expansion and division require higher energy metabolism in WT than in *spm*. In addition, the genes involved in hormone signalling transduction pathway exhibited higher transcriptional activity in WT than in *spm* (Fig. 7B), indicating that plant hormones may be involved in regulating peanut pod size by influencing cell expansion and division.

**Figure 8:**
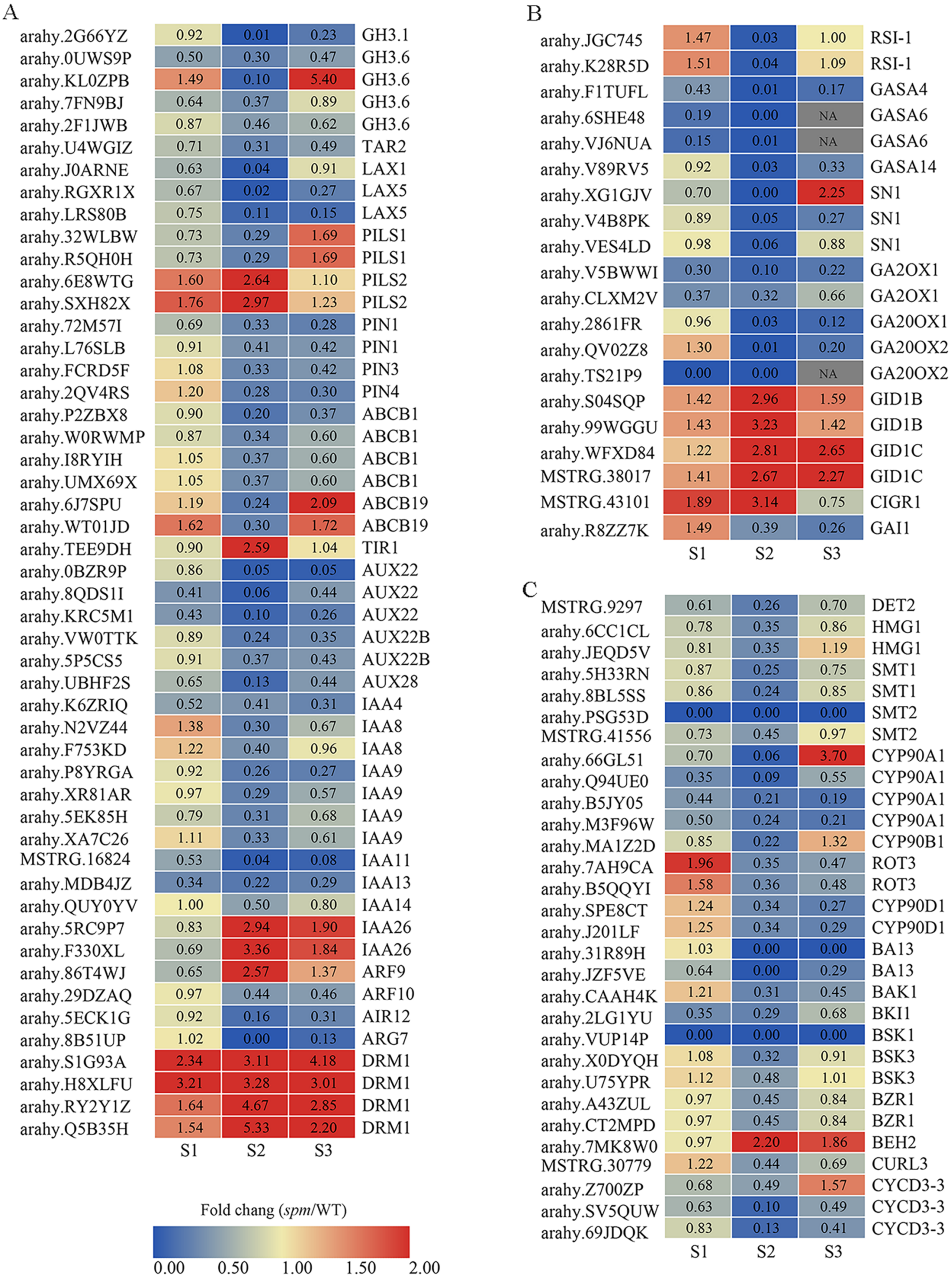
The expression profiles of plant hormones-related genes in *spm* as compared with WT at stages S1–S3. (A) The fold changes (FC) of the auxin-related genes based on the FPKM values in WT and *spm* pods at three developmental stages. (B) The FC of the GA-related DEGs, based on the FPKM in WT and *spm* pods at three developmental stages. (C) The FC of the BR-related DEGs, based on the FPKM in WT and *spm* pods at three developmental stages.

### The expression pattern of cell expansion related genes at stage S2

According to the results of GO analysis, cell expansion-related terms, including ‘cell wall organization or biogenesis’, ‘developmental cell growth’, and ‘cellulose metabolism process’ were significantly enriched at stage S2. In these terms, a total of 48 DEGs encoding xyloglucan endotransglucosylase/hydrolase (XTH), xyloglucan glycosyltransferase (GSLA, GSLC), xyloglucan xylosyltransferase (XXT), cellulose synthase (GESA, GSLD, GSLE, GSLG, and GSLH), and plant invertase/pectin methylesterase inhibitor (PMEI) had significantly lower expression levels in *spm* than in WT at stage S2 (Table S3). These genes are involved in cell wall metabolism, which is crucial for plant cell growth and expansion (Cosgrove, 2005; Liu et al., 2020). In addition, the genes encoding beta-galactosidase 10 (BGAL10), CLIP-associated protein (CLASP), and LONGIFOLIA 2-like protein (LNG2) showed lower expression levels in *spm* than in WT pods. The *BGAL10* gene is responsible for the majority of b-galactosidase activity against xyloglucan, and disruptions of the *BGAL10* cause growth defects and short silique length (Sampedro et al., 2012). The *CLASP* gene encoding a microtubule-associated protein is involved in cell division and expansion (Ambrose et al., 2007). The *LNG2* gene positively regulates longitudinal polar cell elongation in Arabidopsis (Lee et al., 2006).

### The expression pattern of hormones related genes at stage S2

The expression of DEGs related to IAA, GA, and BR showed striking differences at stages S1–S3, especially at stage S2 (Fig. 8). A total of 50 genes exhibited significantly different expression levels in WT and *spm* pods at stage S2 for IAA (Fig. 8A, Table S4). The genes encoding dormancy/auxin-associated family proteins (DRM1), showed significantly higher expression levels in *spm* than in WT at stages S1–S3. The genes encoding protein PIN-LIKES 2 (PILS2) displayed significantly higher expression levels in *spm* than in WT at stages S1 and S2, however, *PILS1* genes exhibited significantly lower transcriptional activity in *spm* than in WT at stages S1 and S2. The expression level of gene encoding protein TRANSPORT INHIBITOR RESPONSE 1 (TIR) was significantly higher in *spm* at stages S2. The genes encoding ARF9 and auxin-responsive proteins (IAA26) exhibited significantly higher transcriptional activity in *spm* at stages S2 and S3, whereas other genes encoding ARF10 and Aux/IAA family members (AUX22, AUX22B, AUX28, IAA4, IAA8, IAA9, IAA11, IAA13, IAA14) showed significantly lower expression levels in *spm* at stages S2 and S3. The expression levels of genes encoding tryptophan aminotransferase-related protein 2 (TAR2), ATP-binding cassette subfamily proteins (ABCB1 and ABCB19), auxin-induced in root cultures protein 12 (AIR12), IAA-amido conjugating enzyme family members (GH3.1 and GH3.6), auxin efflux carrier family proteins (PIN1, PIN3 and PIN4), and auxin transporter-like proteins (LAX1 and LAX5) were significantly higher in WT than in *spm* at stage S2.

A total of 20 genes showed strikingly different expression levels between WT and *spm* pods at stage S2 for GA (Fig. 8B, Table S4). The genes encoding gibberellin 2-oxidase 1 proteins (GA2OX1), GA20OX1 and GA20OX2, and DELLA protein GAI1 (GAI1) showed significantly lower transcriptional activity in *spm* compared with WT at stages S2 and S3. The genes encoding GIBBERELLIN INSENSITIVE DWARF1 (GID1B and GID1C) had higher expression levels in *spm* than in WT at three stages. The genes encoding gibberellin-regulated proteins (GASA4, GASA6, GASA14, and SN1) showed significantly lower expression levels in *spm* than in WT at stage S2.

A total of 30 genes exhibited significantly different expression levels between WT and *spm* pod at stage S2 in BR (Fig. 8C, Table S4). Eleven genes encode cytochrome P450 family proteins (CYP90A1, CYP90B1, ROT3, CYP90D1, BA13). Among them, the genes encoding CYP90A1 and CYP90B1 exhibited lower expression levels in *spm* than in WT at stages S1 and S2. Likewise, the transcriptional activity of genes encoding ROT3, CYP90D1, and BA13 was significantly lower in *spm* at stages S2 and S3. The genes encoding BRASSINAZOLE-RESISTANT 1 proteins (BZR1) showed significantly lower expression levels in *spm* than in WT at three stages, whereas, BES1/BZR1 homolog protein 2 (BEH2) showed significantly higher expression levels in *spm* than in WT at stages S2 and S3. The transcriptional activity of genes encoding BRASSINOSTEROID INSENSITIVE 1-associated receptor kinase 1 (BAK1), BRI1 kinase inhibitor 1 (BKI1), Serine/threonine-protein kinase BSK1 (BSK1), and Brassinosteroid LRR receptor kinase (CURL3) was significantly lower in *spm* than in WT at stages S2 and S3. The genes encoding steroid 5-alpha-reductase DET2 (DET2), 3-hydroxy-3-methylglutaryl-coenzyme A reductase-like proteins (HMG1), sterol methyltransferases (SMT1 and SMT2), serine/threonine-protein kinase BSK3 (BSK3), and CYCLIN D3 (CYCD3-3) exhibited significantly lower expression levels in *spm* than in WT at stage S2.

### Transcription factors (TFs) involved in pod development

We identified a total of 550 TFs (Table S5) at three different developmental stages of peanut pods using the Plant Transcription Factor Database (Jin et al., 2017). Among these TFs, 61 of 98 TFs, 206 of 380 TFs, and 136 of 237 TFs were significantly down-regulated in *spm* compared with WT at stages S1, S2, and S3, respectively. These TFs were classified into 47 gene families (Table 2). The BHLH family was the largest, including 77 members, followed by MYB (38 MYB members and 25 MYB-related members), ERF (34 members), bZIP (32 members), C2H2 (32 members), NAC (31 members) and other TFs families (281 members).

**Table 2:** Transcription factors (TFs) identified in *spm* and WT pods.

TF family	Number of genes	TF family	Number of genes
BHLH	77	C3H	6
MYB	38	CPP	6
MYB-related	25	DBB	5
ERF	34	TCP	5
bZIP	32	ZF-HD	5
C2H2	32	NF-YA	5
NAC	31	Nin-like	5
HD-ZIP	24	SBP	5
WRKY	20	ARR-B	4
Dof	17	RAV	4
HSF	16	WOX	4
LBD	15	BES1	3
Trihelix	14	CO-like	3
G2-like	11	NF-YB	3
B3	10	NF-YC	3
GRAS	10	YABBY	3
FAR1	9	BBR-BPC	2
GRF	9	EIL	2
MIKC	9	LSD	2
TALE	9	HRT-like	1
GATA	8	M-type	1
AP2	7	CAMTA	1
ARF	7	E2F/DP	1
SRS	7	Total	550

### WGCNA analysis and identification hub genes associated with pod size

**Figure 9:**
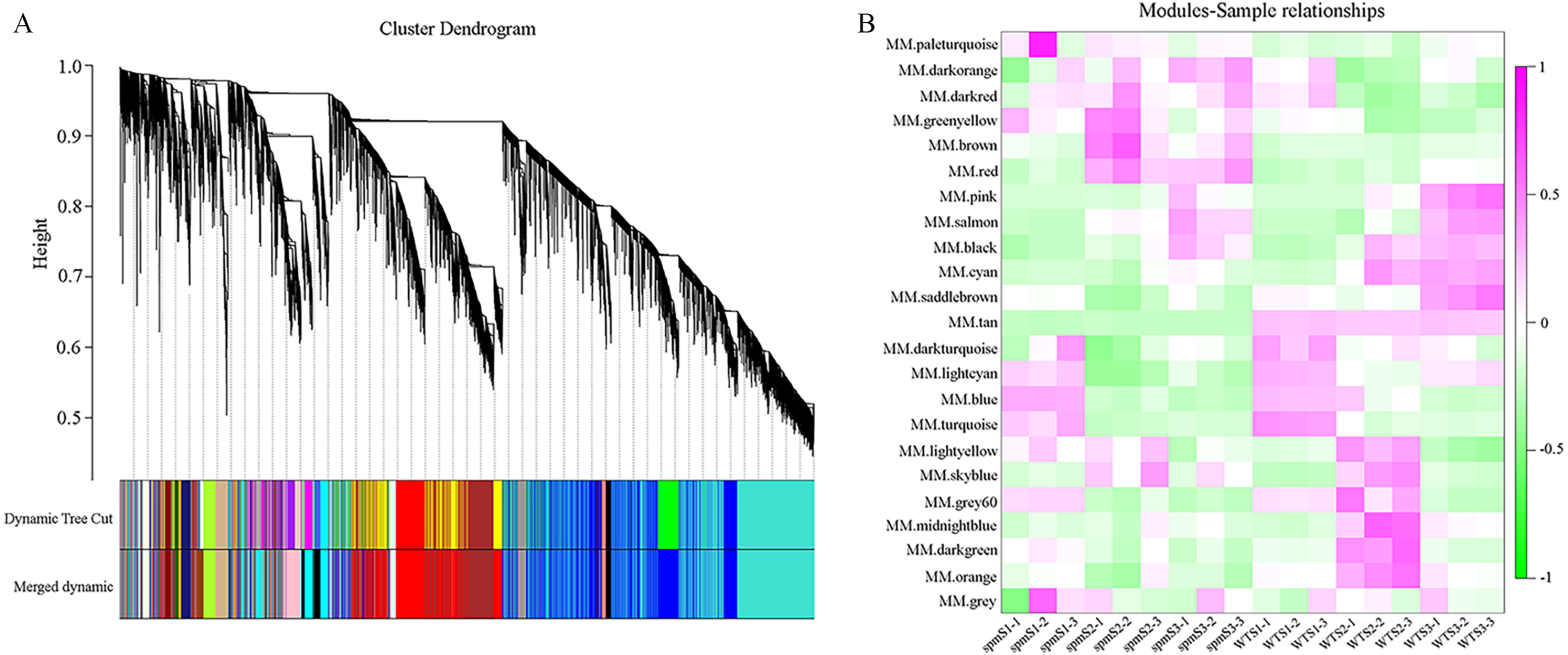
Co-expression network at each pod developmental stage in WT and *spm*. (A) Hierarchical cluster tree of genes based on co-expression network analysis. Each short vertical line in the tree corresponds to one gene. (B) Relationships between co-expressed modules and pod developmental stages in WT and *spm*.

**Figure 10:**
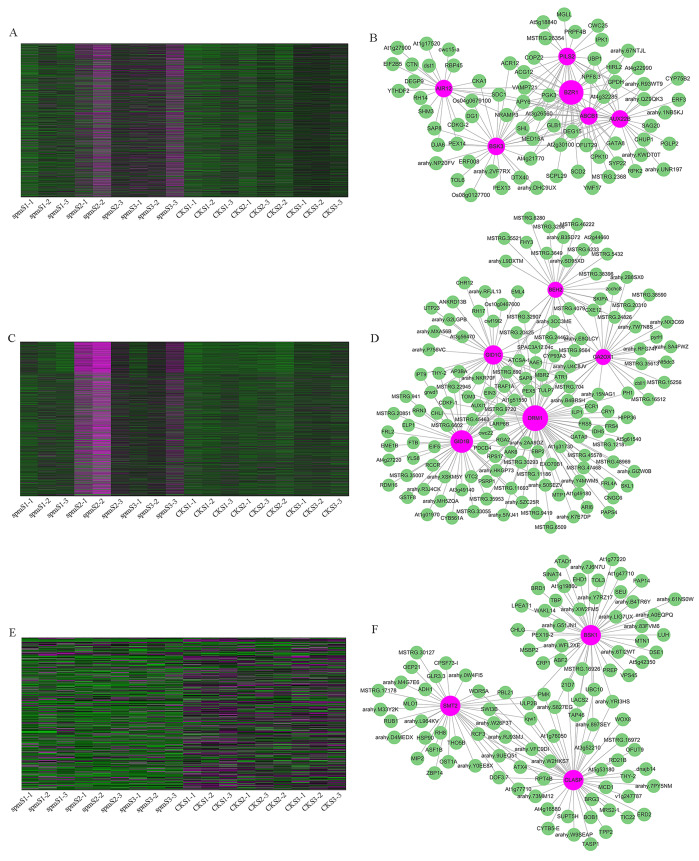
Co-expression network analysis for three modules. (A, C, E) Heatmaps of gene expression patterns for MM.red, MM.brown and MM.tan modules. (B, D, F) The correlation networks for MM.red, MM.brown, and MM.tan modules. Candidate hub genes are shown as red nodes.

WGCNA analysis was performed to explore the gene co-expression regulation network and identify hub genes that were highly associated with peanut pod size. After removing the low expression of genes (FPKM < 0.5), the remaining 30,233 genes were used to perform WGCNA. According to pairwise correlations of gene expression across all samples, a hierarchical clustering tree with 23 co-expression modules was constructed (Fig. 9A). The correlations between modules and different pod developmental stages were investigated. The results showed that the MM.red and MM.brown modules were highly associated with *spm*S2 and *spm*S3, and the MM.tan module was highly associated with all developmental stages of WT pods (Fig. 9B). The expression pattern of the genes in these three modules indicated that some genes may play crucial roles in the regulation of peanut pod size (Figs. 10A, 10C and 10E, Table S6). Therefore, the MM.red, MM.brown and MM.tan modules were selected for gene co-expression network analysis to reveal hub genes related to pod size. Hub genes are those that show the most connections in the network (Du et al., 2017). Forteen genes from these three modules were identified as hub genes when combined with the results of transcriptome analysis (Figs. 10B, 10D and 10F). Among these genes, *DRM1*, *AIR12*, *AUX22B*, *PILS2*, and *ABCB1* are related to auxin transportation or signal transduction pathways; *GIDIB*, *GIDIC*, and *GA2OX1* are associated with GA biosynthesis or signal transduction pathways; *SMT2*, *BEH2*, *BSK3*, *BSK1*, and *BZR1* are associated with BR biosynthesis or signal transduction pathways. These genes showed differentially expressed levels in *spm* and WT pods at different developmental stages (Fig. 8). These results showed that auxin, GA, and BR biosynthesis and signal transduction pathways may be involved in controlling peanut pod size.

### qRT-PCR

Nine hub genes were selected to validate the accuracy of RNA-seq results. These included four genes related to auxin signal transduction pathways, two genes related to GA biosynthesis or signal transduction pathways, and three genes related to BR biosynthesis or signal transduction pathway. The results revealed that the overall expression trend of these nine genes, as quantified by the qRT-PCR, were consistent with those obtained from RNA-seq analysis (Fig. S1), thus demonstrating the reliability of RNA-seq analyses.

## Discussion

Fruit size is an important agronomic trait in crop domestication and improvement (Hussain et al., 2020). Numerous studies in model plants have shown that plant hormones are crucial for determining fruit size by regulating cell expansion and proliferation (Hussain et al., 2020; Fenn & Giovannoni, 2021). However, the mechanism of plant hormones regulating pod size has been not well understood in peanut. In the present study, we performed phenotype analyses for Yuanza 9102 (WT) and its small pod mutant (*spm*) at three stages. The differences in pod size appeared at stage S1, expanded rapidly at stage S2, and peaked at stage S3 (Figs. 1E–1G). As a result, the mature dry pod size in *spm* was strikingly smaller (*P* < 0.001) than in WT (Figs. 1A–1D). Anatomical structure analysis showed that the pod cell sizes were enlarged in both genotypes from stages S1 to S3. However, the cell sizes in *spm* were always smaller than those in WT at three stages, especially at stages S2 and S3 (Figs. 2A–2E). Consistent with the above results, the DEGs related to cell expansion were significantly enriched at stage S2 (Table S3), and most of genes were down-regulated in *spm* compared with WT. Cell size was associated with fruit size (Cheniclet et al., 2005; Liu et al., 2016), therefore, reduced cell size may be one of important factors for small pods in *spm*. Furthermore, the contents of IAA, GA, and BR in *spm* pods were strikingly lower than those in WT at three stages (Figs. 3A–3C). These hormones are essential to regulate growth, specifically cell elongation (Depuydt & Hardtke, 2011). Auxin influences cell division and expansion in developing maize kernels, and auxin deficiency has been shown to result in reduced cell size in the endosperm and a small-seed phenotype (Bernardi et al., 2012; Bernardi et al., 2019). GA reportedly participated in a wide range of physiological processes, including elongation or expansion of numerous tissues of Arabidopsis (Rieu et al., 2008). BR has been shown to be important for cell elongation and division (Planas-Riverola et al., 2019). These results indicated that pod size in peanut may be regulated by auxin, GA, and BR, mainly *via* affecting cell expansion. To our knowledge, ours is the first study to report how plant hormones regulate cell expansion to affect pod size in peanut, which may provide a reference for studying the molecular mechanism of peanut pod size.

Though several major QTLs associated with peanut pod size have been identified, the molecular mechanisms of plant hormones regulation pod size are not well understood. We performed comparative analyses for global transcriptome between *spm* and WT pods at stages S1, S2, and S3. KEGG enrichment analyses revealed that a lot of DEGs from three stages were enriched in plant hormone biosynthesis or signal transduction pathways (Fig. 6C). Pairwise comparisons analysis showed that most of DEGs associated with the plant hormone biosynthesis or signal transduction pathways had significantly lower expression levels in *spm* pods than in WT pods (Fig. 8). Furthermore, the results of WGCNA revealed that several hub genes, including *DRM1*, *AIR12*, *AUX22B*, *PILS2*, *ABCB1*, *GIDIB*, *GIDIC*, *GA2OX1*, *SMT2*, *BEH2*, *BSK3*, *BSK1*, and *BZR1*, were involved in auxin, GA, and BR biosynthesis or signal transduction pathways (Fig. 10). These results showed that the plant hormone biosynthesis and signal transduction pathways may play important roles in regulating pod size in peanut.

Several genes related to auxin biosynthesis or signalling pathways have been reported to affect organ growth and development in model plants (Hussain et al., 2020). *TAR2*, encoding tryptophan aminotransferase-related protein 2, is involved in the auxin biosynthesis pathway (Stepanova et al., 2008). Arabidopsis *TAA1* and *TAR2* double mutation led to multiple organ development deficiencies including dwarfism, reduced vasculature, sterile flowers, and bushy plants with agravitropic roots (Stepanova et al., 2008). Shao et al. (2017) reported that the overexpression of *TaTAR2.1-3A* in wheat increased plant height, spike number, lateral root branching, and grain yield. A loss-of-function mutation in *TAR2*, *tar2-1*, dramatically reduced the content of IAA and seed size in pea (McAdam et al., 2017). Auxin is perceived by a co-receptor complex consisting of TIR1/AFB and Aux/IAA proteins (Hayashi et al., 2008; Prigge et al., 2020). Overexpression of *TIR1* gene (*CsTIR1*) decreased seed size and seeds per fruit in cucumber (Xu et al., 2017). The gene *DRM1*, which belongs to the auxin-repressed superfamily, negatively regulates *Arabidopsis* petioles and siliques size, probably from suppression of either cell expansion or cell elongation (Lee, Han & Hur, 2013). We found that one *TAR2* gene had a significantly lower expression level in *spm* than in WT at stage S2, whereas, the genes *TIR1* and *DRM1*, were significantly up-regulated in *spm* compared with WT at stage S2 (Fig. 8A). These results indicated that *TAR2*, *TIR1*, and *DRM1* may be important regulators for pod size.

Gibberellin is an essential hormone that regulates plant growth and development (Livne & Weiss, 2014). The activity of the *GA20ox* is important for GA production and GA-dependent development. Rieu et al. (2008) reported that *AtGA20ox1* and *AtGA20ox2* promote hypocotyl, internode, and siliques elongation in Arabidopsis, and *ga20ox2* and *ga20ox1 ga20ox2* mutant siliques were significantly shorter than wild type siliques. In this study, the *GA20ox* genes were significantly down-regulated in *spm* compared with WT at stage S2 (Fig. 8B), indicating that *GA20ox* may positively regulate pod size in peanut by mediating GA biosynthesis. BRs, which are steroidal hormones, play important roles in regulating the cell elongation, cell division, and differentiation of various cell types in plant (Li et al., 2018; Planas-Riverola et al., 2019). Campesterol is a biosynthetic precursor of BR (Ohnishi et al., 2006), and loss the function of sterol-related genes results in dysfunctional BR biosynthesis, resulting in dwarfism in Arabidopsis (Schrick et al., 2000; Schaller, 2004). The genes *HMG1* encoding 3-hydroxy-3-methylglutaryl-coenzyme A reductase 1 and *SMT2* encoding 24-methylenesterol C-methyltransferase 2 were involved in sterol biosynthesis. The *hmg1* and *smt2* mutants exhibited short siliques in Arabidopsis (Suzuki et al., 2004; Hwang et al., 2007). In our study, the sterol biosynthesis genes *HMG1*, *SMT1*, and *SMT2* were all down-regulated in *spm* (Fig. 8C). These results suggested that low content of sterol leads to low content of BR, resulting in small pod size in *spm*. As important enzymes in BR biosynthesis, several cytochrome P450 family genes, including *CYP90A1*, *CYP90B1*, *ROT3* and *CYP90D1*, were reported to regulate organ size in Arabidopsis (Kim, Tsukaya & Uchimiya, 1998; Choe et al., 2001; Si et al., 2016). Choe et al. (2001) reported that the overexpression of *DWARF4*/*CYP90B1* resulted in improving BR level and increasing silique length in Arabidopsis. The overexpression of *CYP90A1*/*CPD* and *DWF4*/*CYP90B1*, significantly increased silique length and cell size of cortical in Arabidopsis (Si et al., 2016). The *ROT3* regulated the polar elongation of cells in leaves of Arabidopsis (Kim, Tsukaya & Uchimiya, 1998). *BZR1* is a crucial transcription factor that regulates organ size in the BR signalling pathway in plants (Zhang et al., 2020). The overexpression of *ZmBZR1* led to dramatically larger and longer cells in transgenic *ZmBZR1* lines than in WT plants, and the transgenic *ZmBZR1* lines displayed phenotypes of enlarged cotyledons, floral organs, and seed size in Arabidopsis (Zhang et al., 2020). Overexpression of the *ZmBES1*/*BZR1-5* gene resulted in significantly increase of seed size and weight in rice, and smaller kernels produced in maize mutants (Sun et al., 2021). In our study, several key BR biosynthetic genes *CYP90A1*, *CYP90B1*, *ROT3* and *CYP90D1* were all down-regulated at stage S2 in *spm*, and *BZR1*, the key transcription factor gene in the BR signalling pathway, was down-regulated in *spm* at all stages (Fig. 8C). Therefore, we speculate that *HMG1*, *SMT2*, *CYP90A1*, *CYP90B1*, *ROT3*, *CYP90D1*, and *BZR1* maybe important genes in regulating pod size in peanut.

Plant hormones act synergistically to regulate plant growth and development (Fenn & Giovannoni, 2021). Auxin could increase the expression of *CPY90B1* gene resulting in an enhancement in BR biosynthesis (Chung et al., 2011). In addition, auxin treatment could increase the expression of BR receptor gene *BRI1* leading to an enhancement in BR perception (Saini, Sharma & Pati, 2015). BR also affects polar auxin transport by regulating auxin-responsive genes, such as *PIN2*, and *PIN4* (Hacham et al., 2012). The application of 2, 4-D (a synthetic auxin mimic) could induce *GA20ox* and *GA3ox* expression, and promote cell division and expansion (Cong et al., 2019). The interplay between BR and GA has also been reported (Li & He, 2013; Vanstraelen & Benková, 2012). The BZR1 positively regulates expression of *GA20ox* to induce GA biosynthesis and promote cell elongation (Tong et al., 2014; Unterholzner et al., 2015). These results suggested that significantly down-regulated genes involved in BR/GA biosynthesis and response, and disordered polar auxin transport acted synergistically, leading to small pod size.

## Conclusions

In this study, the pod size and cell size were always smaller in *spm* than in WT at three developmental stages, suggesting that reduced cell size may be one of the important factors for small pod size. A physiological analysis revealed that the contents of IAA, GA, and BR were also lower in *spm* pods than in WT pods at all three stages. We found that many DEGs were associated with cell expansion-related terms and plant hormone signal transduction pathway according to GO and KEGG enrichment analyses. In addition, WGCNA identified several hub genes involved in plant hormone biosynthesis and signal transduction pathways. In summary, the results of our study showed that auxin, GA, and BR may be important regulators in controlling pod size by regulating cell size in peanut. This study promotes our understanding of the complex mechanism of plant hormones in controlling pod size *via* regulating cell size in peanut and may facilitate the improvement of peanut breeding.

## Supplemental Information

10.7717/peerj.12965/supp-1 Figure S1 The qRT-PCR verification of DEGs between *spm* and WT pods from S1 to S3

10.7717/peerj.12965/supp-2 Table S1 The gene-specific primers for qRT-PCR

10.7717/peerj.12965/supp-3 Table S2 The significantly enriched terms in biological processes

10.7717/peerj.12965/supp-4 Table S3 The different expression genes related to cell expansion between WT and *spm* pods at stage 2

10.7717/peerj.12965/supp-5 Table S4 The expression of DEGs related to auxin, GA, and BR showed significant differences between WT and spm pods at stage S2

10.7717/peerj.12965/supp-6 Table S5 TF families were involved in pod development in peanut

10.7717/peerj.12965/supp-7 Table S6 The list of Genes in brown, red, and tan modules

10.7717/peerj.12965/supp-8 Data S1 The raw data for morphological characteristics, anatomy, physiology, and qRT-PCR in WT and *spm* pods

10.7717/peerj.12965/supp-9 Supplemental Information 1 The differentially expressed genes between WT and spm at three stages Pairwise comparison at each developmental stage was performed to investigate the transcriptional differences and a total of 10,396 DEGs (|FC| ≥ 2 and FDR < 0.05) were detected in *spm* pods compared with WT pods.

## Additional Information and Declarations

### Data Availability

The following information was supplied regarding data availability: The RNA-seq raw data are available in the China National Center for Bioinformation: PRJCA006624, GSA: CRA005103. https://ngdc.cncb.ac.cn/gsa/s/56M3N0K4. The pod traits and qRT-PCR data and RNA-seq data are also available as Supplemental Files.
