# Supplementary figures and images for: Transcriptome analysis of pod mutant reveals plant hormones are important regulators in controlling pod size in peanut (Arachis hypogaea L.)
Source: PeerJ. 2022 Feb 28;10:e12965. doi: 10.7717/peerj.12965 (PMC8893032; doi:10.7717/peerj.12965)

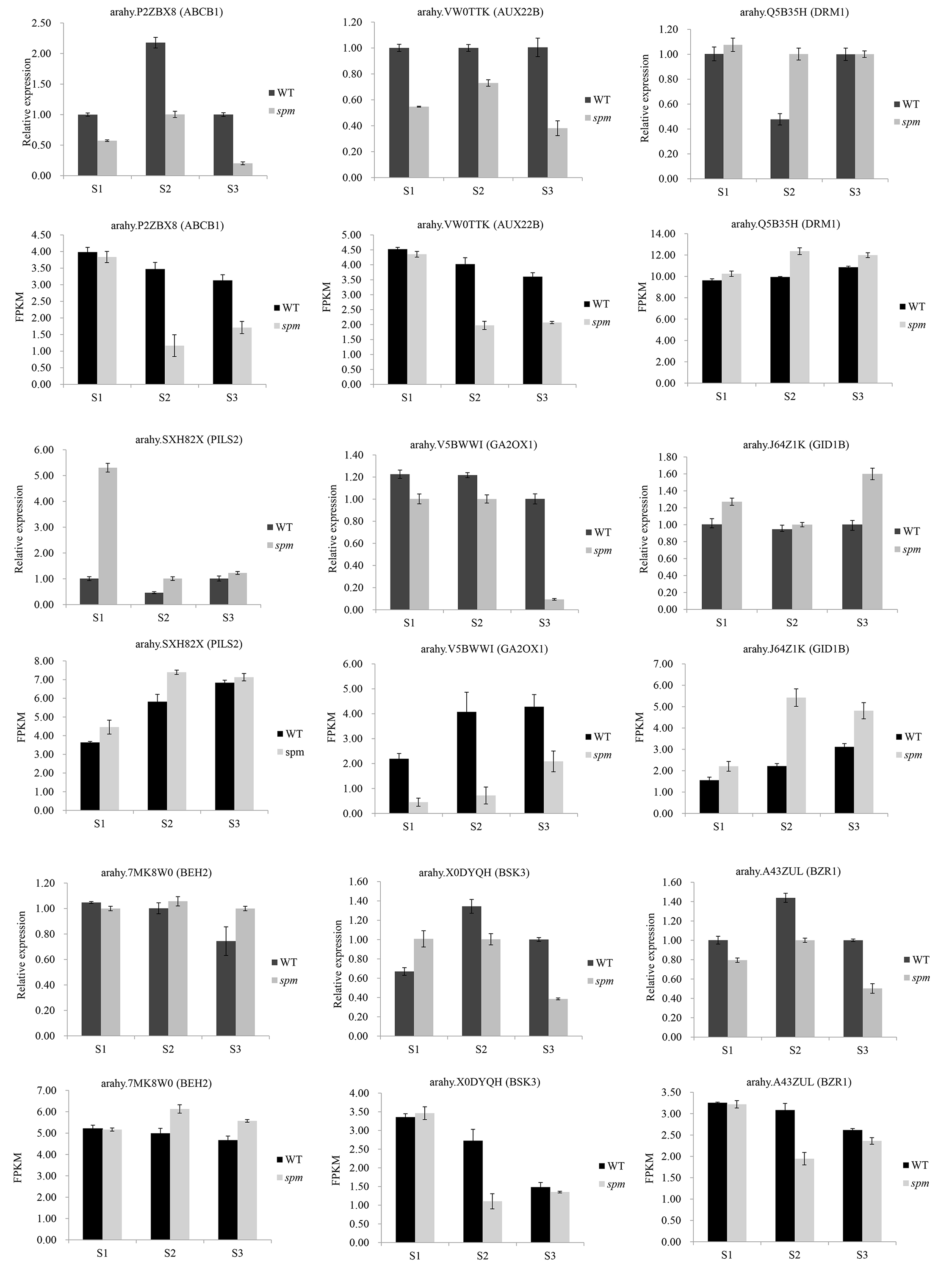

Supplement: Figure S1 [file peerj-10-12965-s001.png]
